# Electron-transfer chain in respiratory complex I

**DOI:** 10.1038/s41598-017-05779-y

**Published:** 2017-07-14

**Authors:** Daniel R. Martin, Dmitry V. Matyushov

**Affiliations:** Department of Physics and School of Molecular Sciences, Arizona State University, PO Box 871504, Tempe, AZ 85287-1504 USA

## Abstract

Complex I is a part of the respiration energy chain converting the redox energy into the cross-membrane proton gradient. The electron-transfer chain of iron-sulfur cofactors within the water-soluble peripheral part of the complex is responsible for the delivery of electrons to the proton pumping subunit. The protein is porous to water penetration and the hydration level of the cofactors changes when the electron is transferred along the chain. High reaction barriers and trapping of the electrons at the iron-sulfur cofactors are prevented by the combination of intense electrostatic noise produced by the protein-water interface with the high density of quantum states in the iron-sulfur clusters caused by spin interactions between paramagnetic iron atoms. The combination of these factors substantially lowers the activation barrier for electron transfer compared to the prediction of the Marcus theory, bringing the rate to the experimentally established range. The unique role of iron-sulfur clusters as electron-transfer cofactors is in merging protein-water fluctuations with quantum-state multiplicity to allow low activation barriers and robust operation. Water plays a vital role in electron transport energetics by electrowetting the cofactors in the chain upon arrival of the electron. A general property of a protein is to violate the fluctuation-dissipation relation through nonergodic sampling of its landscape. High functional efficiency of redox enzymes is a direct consequence of nonergodicity.

## Introduction

One of the most intricate and complex molecular machines evolved to support life is the biological energy chain. It appeared at very early stages of life and has been shared by all three of its domains (archaea, bacteria, and eukaryota)^1^. The molecular complexes responsible for the action are among the largest and most complex membrane proteins known. Yet the action they perform is directed to very small basic subatomic particles of physics: electrons and protons. All energy produced by life is eventually stored in the separation of electrons and protons across the cellular membrane producing the cross-membrane electrostatic potential and the proton motive force^2^. These electrons and protons are driven across the membrane in the opposite directions by the input of energy from light (photosynthesis) or from reducing molecules delivered to mitochondria. This energy input triggers the transfer of electrons, initiating the subsequent proton transfer (4 protons in the case of complex I)^3^.

**Figure 1:**
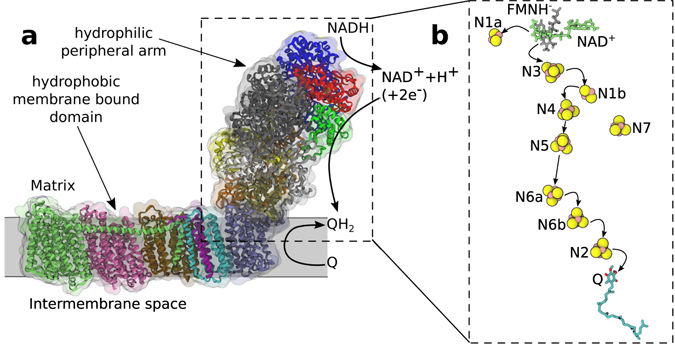
Complex I. (**a**) Peripheral and membrane-bound parts of complex I. (**b**) Cofactors (yellow) involved in the electron transfer chain in the peripheral arm. Also shown are docked NAD^+^ and ubiquinone (Q).

Complex I of mitochondrial or bacterial energy chains exemplifies these design principles^4–7^. It is made of two major parts, the peripheral arm surrounded by the cytoplasm or the mitochondrial matrix and the membrane-bound part^8^ (Fig. 1a). The electron-transfer and proton-transfer pathways are well separated in this complex. Two electrons are injected from NADH docked to the peripheral arm through hydride (H^−^) transfer. They enter the chain of electron transfer cofactors (Fig. 1b) and eventually arrive at ubiquinone/menaquinone at the end of the electron-transfer chain (in the membrane domain), where proton transport across the membrane is initiated.

The electron moves between the cofactors in a sequence of tunneling hops^9^ as discovered by de Vault and Chance^10^. The composition of the chain of cofactors in complex I is unusually uniform and is solely made of iron-sulfur clusters (two Fe_2_S_2_ and seven Fe_4_S_2_ for the *T. thermophilus* structure^6^ adopted here). The resulting profile of the redox potential is essentially flat^3, 5^, with no significant change from the point of electron injection at the docking of NADH to flavin mononucleotide (FMN, Fig. 1b) down to the terminal cofactor N2 with a ~100 meV higher potential^3^. The electron has to undergo a number of elementary hops with very little downhill reaction free energy. The reason for this mechanistic design, and the physical principles making it operate in the robust fashion on the time-scale required for the catalytic turnover (5 ms)^11^, remain unclear. Clarifying the mechanism, physical factors contributing to the reaction barriers, and specific design solutions making such operation robust is the goal of this study.

Fast rate of electron transfer requires balancing two design requirements: maximizing the probability of electron tunneling between the cofactors in the protein matrix^12^ and minimizing the activation barrier to achieve the resonance between the donor and acceptor electronic states required for tunneling. The first requirement is incorporated into the electron-transfer coupling *V* ^2^ ∝ *e* ^−*γr*^ decaying exponentially with the distance *r* between the cofactors^9^. The second component, the free energy of activation Δ*G* ^†^, is typically calculated from the Marcus theory^13^ involving two parameters: the reaction free energy Δ*G*_0_ and the nuclear reorganization (free) energy *λ*

1 $${\rm{\Delta }}{G}^{\dagger }=\frac{{(\lambda +{\rm{\Delta }}{G}_{0})}^{2}}{4\lambda }\mathrm{.}$$

The properties of the protein matrix between the cofactors allowing for the electronic communication between them and the nuclear modes driving thermal agitation to overcome the activation barrier are ultimately the parameters defining the rate of the electron transport, which is given by the Golden Rule expression^14^ (*β* = (*k*_*B*_ *T*)^−1^)

2 $${k}_{{\rm{ET}}}\propto {V}^{2}{e}^{-\beta {\rm{\Delta }}{G}^{\dagger }}\mathrm{.}$$

The question central to the understanding of the design principles of molecular redox machines is how important are the properties of the medium intervening the electron hops. If the protein matrix provides a rather generic tunneling continuum shielding water from the redox centers^15^, the only design variable remaining is the redox potential of the cofactor. If this view is adopted, the pervasiveness of iron-sulfur clusters in energy chains can be credited to their ability to substantially vary their redox potential (in the range of 650 meV^16^), and the corresponding Δ*G*_0_, depending on the binding motif to the protein matrix. The activation barrier is then determined in terms of Δ*G*_0_ and the reorganization energy^15^ for which a generic value of $\lambda \simeq 0.7$–0.8 eV is often assumed^15^. Combined with the generic value of $\gamma \simeq 1.4$ Å^−1^ the equation for the rate suggested by Dutton and co-workers^15^ requires only the input of the reaction free energy to calculate the individual electron-transfer rate.

Neither of these assumptions applies to protein electron transfer. More detailed studies have shown that *γ* depends on the secondary structure and can vary in the range^9, 17, 18^ $\gamma \simeq 1.0$–1.4 Å^−1^. Further, we have shown in the past^19^ and confirm below that no “generic” value of the reorganization energy can be assigned to protein electron transfer and the effective reorganization energy should be used instead. This effective reorganization energy *λ* ^*r*^ (see equation (6) below) is a composite of two reorganization energies, describing the shift and the curvature of the electron transfer parabolas, and can change in a broad range of values.

**Figure 2:**
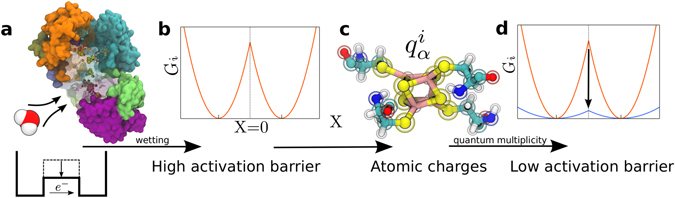
Conceptual framework. (**a**) Wetting of the protein internal pockets lowers the tunneling barrier for electron transfer^21, 22, 24^ (see discussion in the text). (**b**) The activation barrier calculated from MD simulations by using the Marcus theory (equation 1) is too high to be consistent with the experimental transport time of $\simeq 200$ *μ*s. (**c**) Nonergodic electron transfer is introduced within the classical scheme of partial atomic charges interacting with the electrostatic potential created by the protein and water. This formalism requires two reorganization energies, *λ* ^St^ and *λ*, in equation (6). (**d**) The QM/MD formalism allows multiplicity of quantum states and deformation (polarizability) of the electronic distribution of the cofactor in response to electrostatic fluctuations. The vertical arrow indicates the lowering of the activation barrier due to ${\kappa }_{G}\gg 1$ in equations 5 and 6.

The exponential decay of the tunneling probability puts significant restrictions on the design of electron-transfer chains by not allowing efficient electron transfer at edge-to-edge distances exceeding $\simeq \,14$ Å^11, 15^. The charge-transfer chain in complex I fulfills this demand (Fig. 1b). The longest distance, 14.1 Å edge to-edge (~17 Å center-to-center), is between N5 and N6a cofactors, suggesting that this step might be the rate limiting in the entire electron-transfer chain^20^. Hayashi and Stuchebrukhov^21, 22^ studied electron tunneling in complex I and calculated the electron-transfer coupling between N5 and N6a cofactors. Their computed *V* was too low to match the time of electron transport through the chain of cofactors, ~200 *μ*s^20^ (~90 *μ*s was reported in ref. 23). They discovered, however, that allowing structural water to occupy the protein pocket on the electron tunneling pathway dramatically increased *V*, bringing the rate to the experimentally observed range (Fig. 2a). This finding, and similar early calculations^24^, dispel the idea that the intervening medium is generic and not affected by the local structure. The buried protein water can facilitate electronic communication between the cofactors^24^.

These findings are consistent with the recent shift of the view of the role of water in biological function. There is an increasing understanding that water plays an active role in facilitating biological processes^25, 26^, and is also present inside the proteins in amounts not fully appreciated in the past^27, 28^. Water chains are also absolutely essential for the delivery of protons in the proton transport chains^7, 29^. The protein complex performing the redox function should be thus viewed as a medium porous to water penetration. We indeed find here that differential wetting of iron-sulfur cofactors in their different redox states significantly affects the energetics of charge transport in the chain.

The idea that the protein matrix is a porous medium allowing a fairly free exchange of water molecules with the bulk creates some significant conceptual difficulties. The generic value of the reorganization energy^15^ $\lambda \simeq 0.7\mbox{--}0.8$ eV is assigned in the anticipation that the protein provides a nonpolar medium, screening water from direct access to the cofactors. If water is allowed to enter the internal space of the protein, it, being unscreened in confined regions, provides a highly solvating medium. The reorganization energies for redox reactions in aqueous solutions are much higher, at the level of $\lambda \simeq 1.5\mbox{--}2$ eV (or even higher^30^). In addition, water can trap the charge by solvating a certain redox cofactor. This mechanism, related to electrowetting of the protein pocket, prevents back electron transfer between chemically identical quinones in bacterial photosynthesis^31^. Given that iron-sulfur cofactors are essentially equivalent chemically, hydration of each of them has to be very similar to avoid significant reaction free energies. In fact, the distinction between the redox potentials of high-potential iron-sulfur proteins and lower in potential ferredoxins was related to different levels of hydration of the corresponding iron-sulfur clusters^32^. It is difficult to imagine how a generally heterogenous protein matrix can accomplish the requirement of equal hydration and continue the robust operation of the molecular machine. We show below that indeed different levels of hydration, also depending on the redox state, are realized for the redox cofactors in the chain. This observation suggests that new principles for reducing the activation barrier and damping the effect of changes in solvation along the chain have to be found.

We advocate here the view that these new principles have indeed been implemented in natural redox proteins^33^, energy chain complexes, and in complex I specifically. They require a number of new mechanistic ingredients, which alter the rules of establishing the activation barrier for electron transfer from those advocated by the generic Marcus theory^13, 15^ (equation 1). These mechanisms anticipate the universal use, by the protein machines, of the nano-scale electrostatic noise combined with the multiplicity of quantum states of the cofactors. This picture of a noisy, wet interfacial electron transport is quite distinct from the classical view of an orderly progression of redox potentials along the electron transport chain immersed in a solid-like nonpolar protein matrix.

## Results

Among the challenges to understand the mechanistic aspects of the activation barrier for individual electron hops along the chain are the electronic properties of the iron-sulfur cofactors^34^ and their coupling to the fluctuating protein-water thermal bath. In order to understand these issues, we have carried out large-scale molecular dynamics (MD) atomistic simulations of the hydrated peripheral arm of complex I (simulations of the membrane-bound part were recently reported in refs 35 and 35). Our classical MD simulations were combined with quantum calculations of the iron-sulfur, Fe_4_S_4_, cofactors N4, N5, and N6a (Fig. 1b) into a QM/MD valence-bond formalism first developed by Warshel and Weiss^37, 38^.

The Hamiltonian matrix of the QM/MD formalism combines the vacuum eigenenergies of the iron-sulfur cluster with its interaction with the protein-water bath. The interaction enters the diagonal and off-diagonal positions of the matrix (equation (10) below), which is diagonalized at each frame of the simulation trajectory^39, 40^. The instantaneous energies ${E}_{D/A}^{a}$ of the donor and acceptor redox sites (*a* = Red, Ox) are taken as the lowest eigenvalues of this diagonalization and used to produce the fluctuating donor-acceptor energy gap *X* (equation (11) below), which is the reaction coordinate used in modern theories of electron transfer^41–43^. This energy “reaction coordinate”, contrasting more traditional molecular coordinates used in theories of chemical bond breaking/making^44^, specifies the progress of the collective fluctuation of the thermal bath toward the activated state *X* = 0 at which electron tunneling becomes possible. This is the point at which the eigenstates of the donor and acceptor come to resonance as required by Fermi’s Golden Rule (equation (2)).

Combining donor and acceptor quantum eigenvalues with nuclear configurations of the protein-water thermal bath produces the trajectory of the energy gap *X*(*t*). Its statistics, determined by the probability density *P*_*i*_(*X*), depends on whether the electron is localized on the donor (*i* = 1) or on the acceptor (*i* = 2). The free energies of electron transfer *G*_*i*_(*X*) = *G*_0*i*_ − *β* ^−1^ln[*P*_*i*_(*X*)] (Fig. 2b) are determined from the probability densities *P*_*i*_(*X*). The crossing of these surfaces, *G*_1_(0) = *G*_2_(0), at the activated state *X* = 0 provides the activation barrier Δ*G* ^†^ used in the calculations of the rates. More details on the MD simulation protocols and the calculation formalism are given in the Methods below and in the Supplementary Information.

As the first approximation to study the formation of the activation barrier we rely on a more traditional formulation of the Marcus theory of electron transfer implemented in many simulations of synthetic and biological molecules^45–47^. In this approach, which does not impose quantum mechanics overhead on the simulation protocol, quantum calculations of the iron-sulfur cluster in vacuum are used to determine their partial atomic charges ${q}_{\alpha }^{i}$ on the donor and acceptor (Fig. 2c). Two simulation trajectories are produced with charges ${q}_{\alpha }^{1}$ and ${q}_{\alpha }^{2}$. The differences of partial charges in the two states ${\rm{\Delta }}{q}_{\alpha }={q}_{\alpha }^{2}-{q}_{\alpha }^{1}$ are used to produce the electrostatic component of the energy gap *X* ^el^ = ∑_*α*_Δ*q*_*α*_ *ϕ*_*α*_ related to the electrostatic potential of the medium *ϕ*_*α*_ at each atomic site. The statistical analysis of these trajectories results in the free energy surfaces *G*_*i*_(*X*) = *G*_0*i*_ − *β* ^−1^ln[*P*_*i*_(*X*)]. A potential drawback of this approach is that the charge distribution does not relax in response to changes in the environment and overestimates of the electrostatic fluctuations are possible if some polar groups of the environment approach charges Δ*q*_*α*_. To avoid such artifacts, we placed the entire charges of the electron ${\rm{\Delta }}{q}_{D}^{1}=1$ and ${\rm{\Delta }}{q}_{A}^{2}=-1$ at the centers of the corresponding donor (D) and acceptor (A) clusters in our classical calculations based on trajectories obtained with distributed atomic charges. These results are consistent with full quantum calculation when the numbers of excited states of the cluster is reduced and the polarizability of the cluster is neglected (see the Supplementary Information).

Since our sampling of configurations with low probability is limited, the free energy surfaces are parabolas^13^, ${G}_{i}(X)={(X-{X}_{0i})}^{2}\mathrm{/(2}{\sigma }_{X}^{2})$, as expected from the central limit theorem. These parabolas provide two alternative routes to the reorganization energy^33, 42^

3 $${\lambda }^{{\rm{St}}}=|{X}_{01}-{X}_{02}|\mathrm{/2}$$

and

4 $$\lambda ={\sigma }_{X}^{2}\mathrm{/(2}{k}_{{\rm{B}}}T\mathrm{).}$$

The first reorganization energy, *λ* ^St^, reflects the separation of the parabolas’ minima *X*_0*i*_. Since *X*_0*i*_ is the average transition energy, it can be observed when optical charge-transfer bands can be measured along with thermal electron-transfer rates. The value |*X*_01_ − *X*_02_| then becomes the Stokes shift (superscript “St”) separating the absorption and emission maxima in the charge-transfer optical bands. The second reorganization energy, *λ*, quantifies the curvature of the parabolas or, according to the rules of statistical mechanics, the variance of electrostatic fluctuations produced by the medium as described by equation (4). In optical spectroscopy, *λ* quantifies the inhomogeneous broadening of a single vibronic line. Only one reorganization energy is required by the Marcus theory, *λ* ^St^ = *λ*. Based on this assumption, the Stokes reorganization energy is typically reported from simulations since its convergence is much faster than that of *λ* and less demanding simulations are needed^47^.

The restriction of the Marcus theory to only one reorganization energy is often violated in protein electron transfer^33, 46^, for which one observes $\lambda \gg {\lambda }^{{\rm{St}}}$. The distinction between the reorganization energies arising from the first and second statistical moments (equations 3 and 4) is related to nonergodic statistics of the protein-water thermal bath^33^. This phenomenology, often encountered in glass-forming materials^48, 49^, is linked to the inability of the protein to sample its entire configurational space on the time-scale of the reaction. Instead of reaching the global thermodynamic minimum consistent with a given state of the reaction, the protein gets trapped in a metastable state. The result is thermodynamic inconsistency between the first and second statistical moments (*λ* ^St^ and *λ*). This nonergodic sampling can be characterized by the nonergodicity parameter given by the ratio of two reorganization energies

5 $${\kappa }_{G}=\lambda /{\lambda }^{{\rm{St}}}\mathrm{.}$$

The configurational space of a hydrated protein is a complex manifold of many potential energy minima, separated by low barriers, and a smaller number of major basins of stability, further separated by higher activation barriers^50, 51^. Most of the local minima, separated by low barriers, are sampled on the time-scale of 1–100 ns corresponding to elastic deformations altering the protein shape^52^. This elastic thermal agitation, altering the positions of ionized surface residues, produces large-scale fluctuations of the electrostatic potential at the active site of the protein^46^. In contrast, transitions between major basins of stability involve significant conformational changes occurring on a millisecond time-scale^53^. Many of those cannot be reached on the time-scale of the reaction ($\sim 200$ *μ*s considered here).

The consequence of incomplete sampling of the phase space is the breakdown of the fluctuation-dissipation relation^49, 54^, of which the Marcus constraint *λ* ^St^ = *λ* is a special case. If the statistics of *X* is still described by a Gaussian distribution, which is a reasonable expectation from the central-limit theorem, the activation barrier is again described by equation (1) in which the Marcus reorganization energy *λ* is replaced with an effective reaction (superscript “r”) reorganization energy^33^

6 $${\lambda }^{r}={({\lambda }^{{\rm{St}}})}^{2}/\lambda \mathrm{.}$$

**Figure 3:**
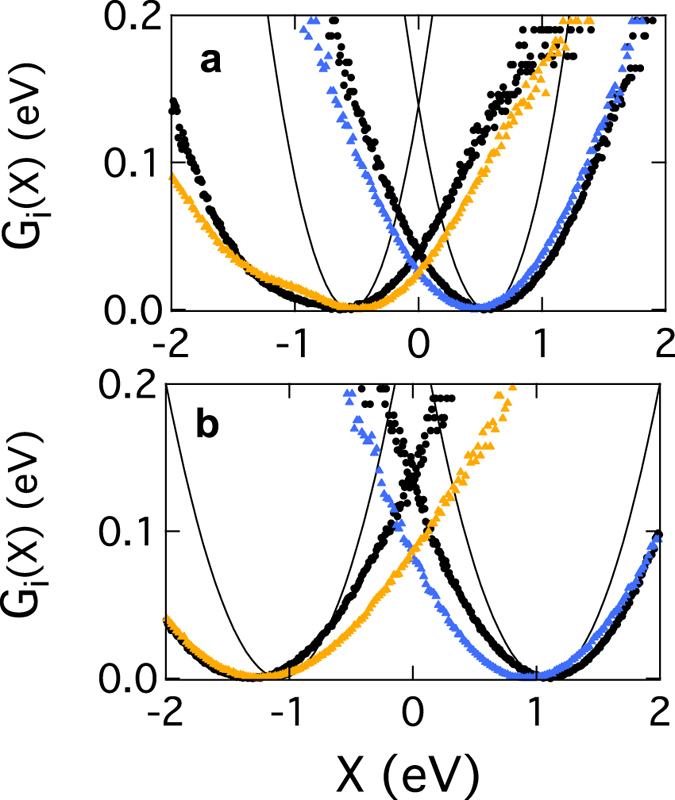
Free energy surfaces. *G*_*i*_(*X*) for electron transfer for N4^−^ → N5 (**a**) and N5^−^ → N6a (**b**). The grey solid lines show the results from the Marcus theory (equation 1) based on *λ* ^St^ from classical MD simulations. The black dots are direct calculations of *G*_*i*_(*X*) from classical MD trajectories. The QM/MD results are shown by the blue points for the forward transition (e.g., N4^−^ → N5) and by the orange points for the backward transition (e.g., N5^−^ → N4).

It is easy to appreciate that the condition ${\kappa }_{G}\gg 1$ results in a significant drop of the barrier compared to the Marcus prediction, as is illustrated for Δ*G*_0_ = 0 in Fig. 3.

Our classical MD simulations show a significant effect of nonergodic sampling on electron transfer. The values of the parameter *κ*_*G*_ obtained in these simulations are listed in Table 1. The result is a substantial lowering of the activation barrier (middle lines in Fig. 3), as also found for bacterial reaction centers and globular redox proteins in solution^33, 46^. In contrast, the Marcus theory leads to a much higher activation barrier and, consequently, the time of N5^−^ → N6a electron transfer ${\tau }_{{\rm{ET}}}={k}_{{\rm{ET}}}^{-1}$ far exceeding the experimental time of electron delivery along the entire chain (Table 1). Changing the reorganization energy from *λ* ^St^ (grey lines in Fig. 3) to *λ* ^*r*^ in equation (6) (black dots in Fig. 3) leads to a dramatic decrease of the activation barrier. The reaction time falls in the experimentally reported range when a higher value of the electron-transfer coupling *V*, involving the structural water in the electron pathway^21^, is used in the calculations (Table 1).

**Table 1:** Reorganization energies^*a*^ and reaction free energies from simulations (eV) and calculated reaction time (*μ*s).

Reaction	*V* ^*b*^	λ^St^	*λ*	κ_*G*_	*τ*_ET_, *μ*s			
					*λ* ^St^ in eq (1)	*λ* ^*r*^ in eq (1)^*c*^	QM/MD	Δ*G*_0_
N4^−^ → N5	3.1	0.6	3.3	6.0	0.3	6.7 × 10^−3^	4.5 × 10^−3^	0.04
N5^−^ → N6a	1.8 × 10^−3^	1.1	3.3	3.1	2.6 × 10^6^	9 × 10^5^	13820	−0.23
	0.05				3611	18	4	

^*a*^The reorganization energies *λ* ^St^ and *λ* from QM/MD simulations. The reported variance reorganization energies *λ* = (*λ*_1 _+ *λ*_2_)/2 are mean values between the forward (*λ*_1_, 1 → 2) and backward (*λ*_2_, 2 → 1) reactions calculated according to equation (4). *λ*_1_/*λ*_2_ are equal to 2.1/4.6 eV for N4^−^ → N5 and 2.7/4.0 eV for N5^−^ → N6a. ^*b*^Electron-transfer coupling (cm^−1^) taken from ref. 22; for N5^−^ → N6a, the smaller value refers to the dry protein and the larger value refers to the protein with structural water included. ^*c*^Determined from classical MD simulations in which *X* = −*e*Δ*ϕ*, Δ*ϕ* is the difference of electrostatic potentials at the centers of the acceptor and the donor.

Wetting of the protein not only enhances the electronic communication between the cofactors. Water plays an active role in driving electron transfer across the activation barrier. Some water molecules are always present in the vicinity of each iron-sulfur cluster, but transferring charge drives changes in the hydration numbers, i.e., it leads to electrowetting of the cofactors^31^. The peripheral arm of complex I is porous to water penetration, and the number of water molecules hydrating the cofactors changes on the time-scale of 100–200 ns following charge transfer (supplementary Fig. S5).

Water also contributes significantly to large-amplitude electrostatic fluctuations experienced by the cofactors. The values of the reorganization energy $\lambda \sim 3$ eV (Supplemental Table S2) are indeed very large compared to what is typically observed for electron transfer in synthetic donor-acceptor systems^14^. The reason for such high intensity electrostatic noise at the protein active site is the concerted character of the interfacial water-protein fluctuations^46, 55^. Elastic deformations of the protein shift both the ionized residues and the domains of surface water polarized by them and attached to the surface by the combination of electrostatic interactions and hydrogen bonds. While strong compensation between the protein and water electrostatic fluctuations is often observed^56^, the resulting *λ* is still very large and often comes as a result of an incomplete compensation between the fluctuations of the electrostatic potential produced by the protein and hydration water. The protein-water interface is, therefore, unique in the molecular world in the way of combining softness of the protein surface (large atomic displacements of wet proteins at high temperatures)^57^ with a high density of surface charge produced by positively and negatively charged residues nearly uniformly distributed over the surface of the protein^58^.

Unique electronic properties of iron-sulfur clusters add to this picture. They possess a high density (multiplicity) of electronic states of different spin quantum numbers, which are energetically accessible through thermal fluctuations^21^. This property suggests that these clusters can switch between many adiabatic electronic states in the course of a medium fluctuation bringing the system to the activated state of electron tunneling (*X* = 0). Such multiplicity of quantum states adds to high intensity, nonergodic electrostatic fluctuations of the protein-water interface in bringing the activation barrier down. An additional reduction of the free energy required to arrive at the activated state comes from the ability of the system to relax by switching to a different electronic state. A part of our agenda here is to explore this mechanism in an attempt to address the question of why iron-sulfur cluster are so ubiquitous in protein redox chemistry.

The energies of the donor and acceptor states entering the energy-gap reaction coordinate *X* (equation (11) below) come from diagonalizing the Hamiltonian of the iron-sulfur cluster involved 100 excited states for each spin configuration of the cluster (see Methods below and Supplementary Information). A multiplicity of quantum states between which the system can switch physically implies its high electronic polarizability (deformability of the electronic density). A polarizable quantum center carrying the polarizability *α*_0_ gives rise to the polarization free energy −(1/2)***E***_*b*_ ⋅ *α*_0_ ⋅ **E**_*b*_, where **E**_*b*_ is the electric field of the thermal bath. On the other hand, the free energy (reversible work) invested in creating a fluctuation in the medium scales quadratically with the field, $(\chi \mathrm{/2)}{{\bf{E}}}_{b}^{2}$. The addition of the polarization free energy to this term lowers the force constant for the medium fluctuation from *χ* to $\simeq (\chi -{\alpha }_{0})$. When projected on the reaction coordinate of electron transfer *X*, less free energy invested in an electrostatic medium fluctuation implies lower curvature of the corresponding parabola *G*_*i*_(*X*), as we indeed observe for the free energy curves (Fig. 3).

**Figure 4:**
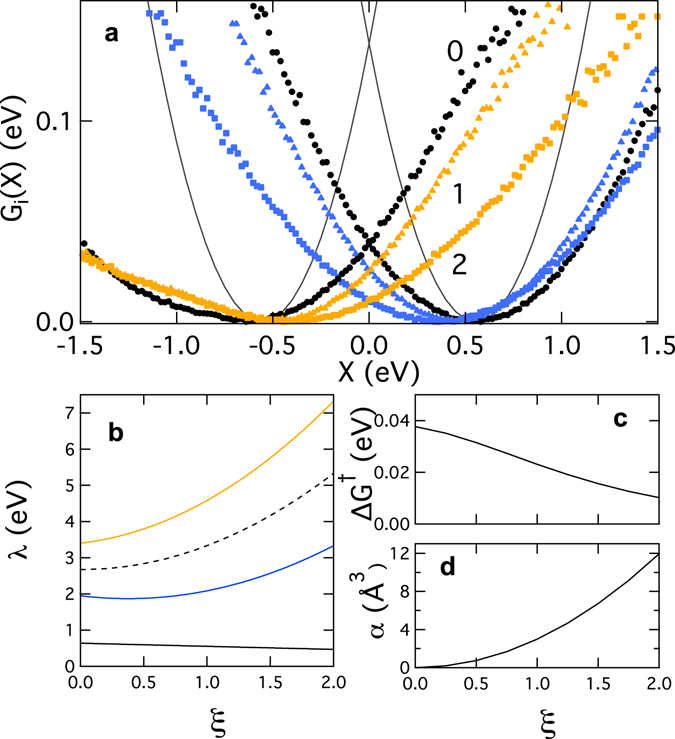
Effect of polarizability. The transition dipoles in the Hamiltonian matrix were scaled, *μ*_*jk*_ → *ξμ*_*jk*_, to determine the effect of the cofactor polarizability on the energetics of the forward N4^−^ → N5 (blue) and backward N5^−^ → N4 (orange) transitions. (**a**) Free energy surfaces at *ξ* = 0 (black, solid lines indicate the Marcus theory), *ξ* = 1, and *ξ* = 2 (marked in the plot). (**b**) *λ*_1_ (N4^−^ → N5, blue) *λ*_2_ (N5^−^ → N4, orange), and their mean (dashed black). The Stokes shift reorganization energy *λ* ^St^ is marked solid black. (**c**) The activation barrier Δ*G* ^†^. (**d**) The polarizability of N4 in the ground-state spin state.

Molecular polarizability is a property slowly converging as a function of the number of excited states (Supplemental Figs S3 and S4). In addition, the transition dipoles provided by the standard quantum packages do not include the effects of high-energy excited states^59^. Both effects contribute to a potentially underestimated values of the cofactor polarizability. Upward rescaling of our calculated set of transition dipoles, *μ*_*jk*_ → *ξμ*_*jk*_, increases the polarizability (Fig. 4d) and drives the activation barrier further down (Fig. 4a,c). We also note that our calculations of the rates neglect the fact that the reorganization energies *λ*_1_ for the forward transition is distinct from *λ*_2_ for the backward transition (Fig. 4b). The numbers listed in Table 1 are mean values *λ* = (*λ*_1_ + *λ*_2_)/2 (dashed black line in Fig. 4b).

The rate constant requires the free energy of the reaction Δ*G*_0_ (equations (1) and (2)), which can be calculated from a modification of the Rehm-Weller equation^60^ (see Supplementary Sec. 2 for the derivation)

7 $${\rm{\Delta }}{G}_{0}={\rm{\Delta }}{G}_{{\rm{redox}}}+{W}^{C}\mathrm{.}$$

Here, ${\rm{\Delta }}{G}_{{\rm{redox}}}=e({E}_{m}^{D}-{E}_{m}^{A})$ is the difference of the midpoint potentials of the donor and acceptor, ${E}_{m}^{D,A}$ (oxidation for the donor and reduction for the acceptor). The Coulomb correction *W* ^*C*^ is traditionally sought from the relation^60^

8 $${W}^{C}=-\frac{{e}^{2}}{{\varepsilon }_{{\rm{eff}}}{R}_{DA}}({Z}_{D}^{{\rm{Ox}}}-{Z}_{A}^{{\rm{Ox}}}),$$

where ${Z}_{D}^{{\rm{Ox}}}$ and ${Z}_{A}^{{\rm{Ox}}}$ are the charges of the oxidized states of the donor and acceptor, *R*_*DA*_ is the donor-acceptor distance, and *ε*_eff_ is the effective dielectric constant of the intervening medium. Equation 8 is not very practical since the dielectric constant *ε*_eff_ is not precisely known^61^ and, in addition, the dielectric formalism used in the derivation is not well suited to the heterogeneous protein-water interface. An alternative to this relation can be obtained (see Supplementary Information) in terms of the correlation function 〈*δϕ*_*D*_ *δϕ*_*A*_〉 between the fluctuations of the electrostatic potential created by the medium at the donor, *δϕ*_*D*_, and the acceptor, *δϕ*_*A*_

9 $${W}^{C}=-{e}^{2}({Z}_{D}^{{\rm{Ox}}}-{Z}_{A}^{{\rm{Ox}}})[{R}_{DA}^{-1}-\beta \langle \delta {\varphi }_{D}\delta {\varphi }_{A}\rangle ]\mathrm{.}$$

**Figure 5:**
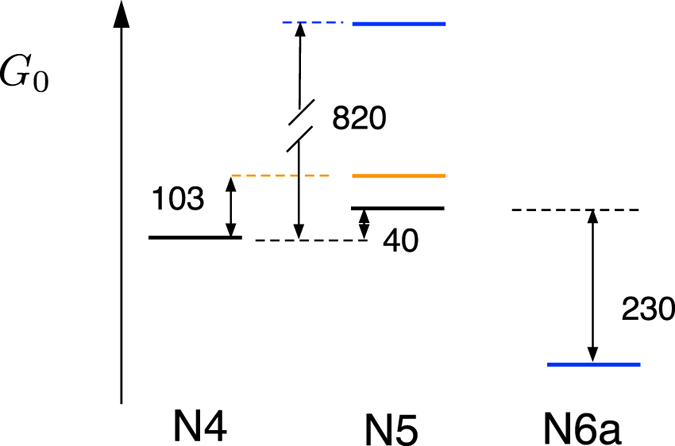
Free energy profile. Δ*G*_0_ calculated from equation (7) assuming Δ*G*_redox_ = 0. The values of Δ*G*_0_ are shown in meV for electron on N4 (black), on N5 (blue), and on N6a (orange). The transition of the second electron to N5^−^ is prohibited by a very high uphill free energy of $\sim 820$ meV.

The main qualitative result of the calculation based on equations (7) and (9) is the appearance of a step in Δ*G*_0_ separating N4 and N6a redox states (Fig. 5). The cofactors in the chain are believed to be equipotential, with possible variations in Δ*G*_redox_ not exceeding $\sim 30$ meV^3^. However, ligation of N5 by histidine makes it different from other iron-sulfur cofactors ligated by cysteine and results in its oxidized charge ${Z}_{N5}^{{\rm{Ox}}}=-1$, more positive than ${Z}_{N4}^{{\rm{Ox}}}={Z}_{N6a}^{{\rm{Ox}}}=-2$ for N4 and N6a. This change in ligation makes $\Delta Z={Z}_{D}^{{\rm{Ox}}}-{Z}_{A}^{{\rm{Ox}}}$ in equation (9) negative, Δ*Z* = −1, for the N4^−^ → N5 reaction and positive, Δ*Z* = +1, for the N5^−^ → N6a reaction. Correspondingly, the Coulomb correction *W* ^*C*^ in equations (7) and (9) yields a positive uphill free energy ${\rm{\Delta }}{G}_{0}\simeq 40$ meV for the N4  →  N5 transition and a negative downhill ${\rm{\Delta }}{G}_{0}\simeq -230$ meV for the N5  →  N6a transition (assuming Δ*G*_redox_ = 0, see Supplemental Table S4 for the potential cross-correlations). The change in ligation, the reasons for which would be otherwise hard to understand, thus slows down the N4^−^ → N5 reaction and accelerates the N5^−^ → N6a reaction. The reaction time for the former is $\sim 5$ ns (Table 1). The uphill step of $\sim 40$ meV and the corresponding slowing down do not affect the overall performance of the charge-transfer chain requiring 90−200 *μ*s for all reactions within the chain. On the contrary, the N5^−^ → N6a reaction is likely the rate limiting step, and the reaction with Δ*G*_0_ = 0 has the reaction time of $\sim 86$ *μ*s, potentially challenging the overall performance. Producing a downhill reaction free energy of $\sim 200$ meV brings the reaction time down to 4 *μ*s, thus avoiding kinetic constraints on the overall performance.

Along the same lines, hydration of N5^−^ following electron transfer lifts the reaction free energy to ${\rm{\Delta }}{G}_{0}\sim 820$ meV. The second electron cannot arrive to N5 and the iron-sulfur cluster remains a one-electron redox cofactor. A higher level of the free energy is partially preserved also for the reduced N6a^−^ state (orange in Fig. 5). The reaction free energy for the N4^−^ → N5 transition is ${\rm{\Delta }}{G}_{0}\simeq 100$ meV for the N6a^−^ state. This observation implies that reducing N6a prevents populating N5. This phenomenology is consistent with experiment: electrons distribute onto alternating clusters in the chain^62^. The explanation for this behavior was originally sought in the picture of exergonic and endergonic steps in the redox potentials often observed for electron transport chains^15, 63^. Our calculations suggest that alternating electron population is caused by the Coulomb correction *W* ^*C*^ in the reaction free energy, which is influenced by electrowetting of cofactors in response to the arrival of electron. In the final state N6a^−^, the average number of waters increases on the time-scale of $\sim 150$ ns from nearly 0 to $\sim 6$ waters (Supplemental Fig. S5). This increase in hydration leads to an increase in fluctuations of the electrostatic potential at N6a. Accordingly, the fluctuation reorganization energy *λ* (equation (4)) charges from $\simeq 2.7$ eV for the N5^−^ → N6a reaction to $\simeq 4$ eV for the N6a^−^ → N5 reaction (Supplemental Table S2). Since the time of electrowetting is shorter than the reaction time, hydration of the N6a cofactor creates an uphill free energy step of $\sim 300$ meV for the backward N6a^−^ → N5 transition (from blue to orange lines in Fig. 5). Wetting of the protein produces no major conformational change, except for a ~0.8 Å decrease in the separation of the cofactors when N6a is reduced (Supplemental Table S1).

## Discussion

Computer simulations performed here have shown that the combination of polar and charged groups of the protein matrix with the penetration of water into confined regions of the protein produce a highly polar, wet environment for electron-transfer transitions. Large solvation energies caused by such media would trap the electron at the chain sites and effectively cease electron transport if the standard theories are applied. The resolution of this dilemma is found in the combination of high intensity electrostatic fluctuations, produced by the protein-water interface, with quantum-state multiplicity of the iron-sulfur cofactors resulting in deformability of their electronic clouds by the medium (electronic polarizability). These physical factors combine with the generic glassiness of protein’s exploration of its energy landscape characterized by a large number of local traps. Such traps do not allow the protein to reach its global minimum consistent with a given electronic state of the cofactor. The result is the violation of the fluctuation-dissipation theorem^49, 54^ causing high functional efficiency of redox enzymes^33^.

The mechanistic consequence of the statistical properties of the protein-water thermal bath relevant to electron transfer is a strong separation, $\lambda \gg {\lambda }^{{\rm{St}}}$, between the Stokes and variance reorganization energies and the resulting small value of *λ* ^*r*^ (equation (6)). These physical factors lower the activation barrier for the N4^−^ → N5 reaction to a value of the order of $\sim {k}_{{\rm{B}}}T$. For N5^−^ → N6a, the barrier is higher, but the calculations performed here produce the reaction time ${\tau }_{{\rm{ET}}}\sim 4$ *μ*s, below the reported time of electron transport in the chain, ~(90–200) *μ*s^3, 20^. However, our calculations also demonstrate that the design challenges of keeping a sufficiently fast electron flow in the chain can be substantial and the number of cofactors in the chain is kept right below the threshold of the chain becoming too slow. The longest tunneling distance between N5 and N6a drives the reaction close to the performance threshold ($\sim 86$ *μ*s at Δ*G*_0_ = 0). This potential bottleneck is eliminated by altering cysteine to histidine ligation of the N5 cofactor, which changes its charge and allows a negative free energy of this rate limiting reaction (Fig. 5).

From a general perspective, protein electron transfer is far less generic than has often been assumed. Local structure of the protein exposed to various levels of wetting by water demands specific design solutions to both allow a unidirectional flow of electrons and the robust operation not affected by small variations in the external conditions. A high level of the electrostatic noise of the protein-water interface effectively washes out small alterations in the operation conditions and effects of insignificant mutations. Operation in the high-amplitude electrostatic noise of the protein-water thermal bath provides the resolution of the robustness dilemma since the sensitivity of the cofactors to small changes in the environment is reduced by the noise. Intrinsic nonergodicity of this noise, combined with quantum multiplicity of the cofactors, lowers the barriers for electronic transitions, while still keeping it high for the undesired side reactions. This latter feature provides vectorial electron transport along the chain of redox cofactors. Wetting of specific sites (N6a here) following electronic transitions offers the mechanism of electrowetting, which prevents back reactions and allows a unidirectional flow of the electrons.

## Methods

This paper describes fully atomistic MD simulations of the peripheral arm of complex I (37427 atoms) hydrated by 147960 TIP3P water. The total simulation length, including all charge states of the iron-sulfur cofactors, was 1.129 *μ*s. Simulations were performed using NAMD 2.9^64^ with CHARMM36 (with CMAP torsion corrections) force field^65^ applied to the protein and NAD^+^. Separate DFT calculations of the atomic charges of the protonated FMN cofactor were done as described in more detail in the Supplementary Information.

The QM component of the analysis was performed by expanding the electrostatic potential of the bath *ϕ*(**r**) around the potential *ϕ*_Fe_ calculated as the average of potentials at four iron ions. The multipolar expansion is truncated at the dipolar term. The matrix elements of the cofactor’s Hamiltonian become^39, 40^

10 $${H}_{jk}^{a}=({E}_{j}^{a}+{Q}^{a}{\varphi }_{{\rm{Fe}}}){\delta }_{jk}-{\mu }_{jk}^{a}\cdot {{\bf{E}}}_{b}\mathrm{.}$$

Here, *E*_*j*_ is the energy of the vacuum eigenstate *j*, *Q* ^*a*^ is the total charge of the iron sulfur cluster, and ${\mu }_{jk}^{a}$ is the transition dipole between the *j*th and *k*th quantum states. The index *a* = {Red, Ox} specifies the redox state of the cluster. Finally, **E**_*b*_ is the electric field of the medium calculated as the average over the iron atoms. The minimum eigenvalue obtained by diagonalizing the Hamiltonian for each spin configuration of the donor and acceptor produce four instantaneous ground-state energies ${E}_{g}^{a}(A)$ and ${E}_{g}^{a}(D)$. They are combined in the instantaneous energy gap^41, 43^

11 $$X=[{E}_{g}^{{\rm{Ox}}}(D)-{E}_{g}^{{\rm{Red}}}(D)]-[{E}_{g}^{{\rm{Ox}}}(A)-{E}_{g}^{{\rm{Red}}}(A)]\mathrm{.}$$

This energy gap is sampled along the trajectory to produce the corresponding free energy surfaces from the normalized distribution functions *P*_*i*_(*X*)

12 $${G}_{i}(X)={G}_{0i}-{\beta }^{-1}{\rm{l}}{\rm{n}}[{P}_{i}(X)],$$

where *i* = 1 denotes the forward transition and *i* = 2 denotes the backward transition; Δ*G*_0_ = *G*_02_ − *G*_01_. Given that the distribution functions are normalized, our calculations do not give access to the reaction free energy Δ*G*_0_. It was calculated from equations (7) and (9) assuming Δ*G*_redox_ = 0 (Table 1). Finally, the nonadiabatic rates were calculated as^14^

13 $${k}_{{\rm{ET}}}=\frac{{V}^{2}}{\hslash }{(\frac{\pi \beta }{{\lambda }^{r}})}^{\mathrm{1/2}}{\rm{e}}{\rm{x}}{\rm{p}}[-\beta \frac{{({\lambda }^{r}+{\rm{\Delta }}{G}_{0})}^{2}}{4{\lambda }^{r}}]\mathrm{.}$$

The reaction times listed in Table 1 are ${\tau }_{{\rm{ET}}}={k}_{{\rm{ET}}}^{-1}$.

Coupling of high-spin iron atoms in the iron-sulfur cluster creates unique physics of electron delocalization between the pairs of ferromagnetically coupled iron atoms due to the double-exchange interaction. Additional electron density delocalization occurs via the sulfur ligands bridging the antiferromagnetically coupled layers in the cluster^66^ (which are better electron donors than terminal thiolates). The arising multitude of quasi-degenerate energy levels is responsible for a high density of states^22^ within the characteristic width, (2*λk*_*B*_ *T*)^1/2^, of electrostatic fluctuations. Additional mixing of electronic states can potentially occur through thermally activated vibrational dynamics^67^. This component is currently missing from our analysis.

The antiferromagnetically coupled spin states of the cluster require broken-symmetry calculations as described in more detail in the Supplementary Information. Each broken-symmetry spin configuration of the cluster was separately calculated at the ZINDO level^21, 22^, including 100 excited states (CIS calculations with 50 excited states were also attempted, see Supplemental Table S3). These 100 states form the 100 × 100 block of the entire Hamiltonian matrix composed of all possible spin states of the cluster (e.g., 24 for the reduced Fe_4_S_4_, Supplemental Fig. S2). The Hamiltonian matrix was then block-diagonalized and the lowest energy among the eigenvalues from all blocks was chosen for ${E}_{g}^{a}({\rm{A}}/{\rm{D}})$. This procedure was repeated for each configuration along the MD trajectory. A single spin state from the manifold of spin configurations typically dominates in the trajectory. The variance of the energy gap is mostly a sum of the corresponding variances at the donor and acceptor sites (see Supplemental Table S2 for cross correlations). In this sum, the variance of the fluctuations at the more hydrated cofactor (notably N6a^−^) typically dominates in the total.

### Data Availability

The simulation data produced during the current study are available from the corresponding author upon request.

## Electronic supplementary material

Supplementary Information
