# Supplementary material for: Electron-transfer chain in respiratory complex I
Source: Sci Rep. 2017 Jul 14;7:5495. doi: 10.1038/s41598-017-05779-y (PMC5511282; doi:10.1038/s41598-017-05779-y)
Supplement: Supplementary file 1 — Supplementary Information [file 41598_2017_5779_MOESM1_ESM.pdf]

Electron-transfer chain in respiratory complex I: Supplementary information

Daniel R. Martin[1, a)] and Dmitry V. Matyushov[1, b)]
Department of Physics and School of Molecular Sciences, Arizona State University, PO Box 871504, Tempe, Arizona 85287

I. Molecular dynamics protocol

A. System setup

The initial coordinates used for all simulations of the hydrophilic peripheral arm of complex I (NADH:ubiquinone oxidoreductase) were adopted from the Protein Data Bank entry 3IAM and correspond to bacterium *T. thermophilus*.[1] The original PDB file contains eight different protein subunits, a 1,4-dihydronicotinamide adenine dinucleotide (NAD) molecule, a flavin mononucleotide (FMN), and nine iron-sulfur clusters (ISCs). These cofactors include two Fe_2S_2 (N1a, N1b) and seven Fe_4S_4 (N2, N3, N4, N5, N6a, N6b, N7) ISCs. The topological structure was created by separating all subunits, individual molecules, and cofactors and recombining with the addition of hydrogen atoms and application of all force-field parameters using the VMD subprogram `psfgen`.[2] The force field topology and parameters for the protein subunits were taken directly from the CHARMM36 (with CMAP torsion corrections) force field.[3] CHARMM force field parameters for neutral FMN were taken from studies of the flavin-binding LOV (Light-Oxygen-Voltage) domains in the phototropin receptor protein.[4,5] CHARMM parameters for both NADH and NAD^+ have previously been published and the parameters corresponding to NAD^+ were applied here.[6,7]

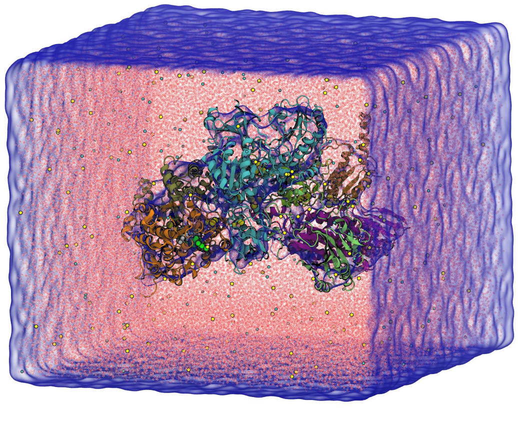

FIG. S1. Simulation box with the hydrophilic part of complex I.

a)Electronic mail: daniel.martin@asu.edu
b)Electronic mail: dmitrym@asu.edu

The parameters for the iron-sulfur clusters (including modified ligated protein amino acids) were taken directly from DFT calculations performed by Chang and Kim.[8]

The electron transfer reactions between the ISC cofactors follow the hydride transfer from NADH to FMN. We therefore considered the protonated form of FMN (FMNH) and deprotonated form of NADH (NAD^+). The new charges for the FMNH were calculated with DFT (B3LYP/6-311+G) implemented in Gaussian09. A single hydrogen atom was added to the N5 nitrogen of the flavin and the ribitol alcohol was removed and replaced by a single hydrogen atom (closed shell). Following the geometry optimization, the partial atomic charges were obtained from a fit to electrostatic potential (ESP) using the "CHelp" option.[9] Once the charges were obtained, the ribitol alcohol was added back to the structure and the residual charge from the extra hydrogen was added back to the corresponding carbon atom to assure unit charge on both the flavin and ribitol substructures.

The ISC cofactors were attached to the protein matrix through applied patches using `psfgen` for all corresponding ligations, with the patches obtained from the supplemental material provided by Chang and Kim.[8] These patches include both oxidized and reduced charge states for all ISCs plus ligands, along with bond, angle, and dihedral force field parameters.

Finally, after all the charges and topological information (bonds, angles, dihedrals, improper angles, etc) were applied to complex I, TIP3P water was added to the system using the CHARMM force field and the `solvate` plugin from VMD. Ions were added to the system using the `autoionize` plugin, with the resulting ionic concentration of $\simeq$50 mM/L. The resulting system consisted of 481641 atoms in an orthorhombic box with x, y, z box lengths of $178.9 \times 148.3 \times 178.1$ Å(Fig. S1). The box dimensions were taken after a short 20 ns NPT simulation (details below) and held constant throughout all subsequent NVT simulations.

B. Simulation protocol

All simulations were performed using NAMD 2.9.[10] The initial structure described in the previous section was first simulated (NPT) for 20 ns in order to relax the waters surrounding the complex I with all ISCs in their oxidized states except for the reduced N4. This was followed by 5000 steps of steepest decent minimization. This initial equilibration was followed by 340 ns of production for the charge transfer state $N4^-$/N5/N6a, with the electron on N4, using the NVT ensemble. The starting configuration for the charge state N4/$N5^-$/N6a was extracted at the 60th ns of the previous run. The new charges were then applied and a 328 ns NVT simulation was carried out. Finally, the configuration after

TABLE S1. Distances (Å) and rms displacements (Å^2) between the cofactors in the chain obtained from MD simulations.

State	$\langle d_{I,II}\rangle$	$\langle \delta d^2_{I,II}\rangle$	$\langle d_{II,III}\rangle$	$\langle \delta d^2_{II,III}\rangle$	$\langle d_{I,III}\rangle$	$\langle \delta d^2_{I,III}\rangle$
N4$^-$ (I)	12.56	0.05	17.00	0.04	28.49	0.10
N5$^-$ (II)	12.98	0.10	17.33	0.05	29.01	0.39
N6a$^-$ (III)	12.71	0.03	16.52	0.26	27.66	0.27

100 ns simulation of the N4/N5$^-$/N6a charge state was used as the starting configuration for the final simulation of the charge state N4/N5/N6a$^-$ simulated for 461 ns. The total simulation length, including all charge states, was 1.129 μs.

All production simulations were done using the NVT ensemble with the Langevin temperature control set to 300 K and a damping coefficient of 5 ps^{-1}. Long-range electrostatic interactions were treated with the particle mesh Ewald technique using a cutoff distance of 12.0 Å. A 2.0 fs timestep was used for all simulations, along with a 1 ps saving frequency, except for the initial 60 ns of the charge state N4$^-$/N5/N6a for which the saving frequency was 2 ps. It was noticed that after approximately 50 ns, some of the ISCs would collapse resulting in unphysical geometries due to a new minimum in the angle and dihedral energies that was not offset by the van der Waals interactions. For this reason, we increased the problematic angle and dihedral force constants (making the structures slightly more rigid) incrementally making it impossible for the ISCs to populate these unwanted and unphysical geometries and assuring the stability of the simulations.

The center-to-center distances between the cofactors obtained from MD simulations are listed in Table S1. The protein matrix does not show major conformations in response to moving electrons. However, the hydration levels of N5 and N6a cofactors change upon the arrival of the electron (see below). In addition, there is a shortening of the distance between the cofactors in the reduced N6a$^-$ state.

C. Quantum calculations

The coordinates of a particular iron-sulfur cluster were extracted from the PDB file (PDB:3IAM[1]) and used as the starting point for the quantum mechanical (QM) calculations. All QM calculations were done with Gaussian 09.[9] The original cysteine (and histidine) residues were removed from the entire ligated irons-sulfur clusters, keeping only the organic sulfur (or nitrogen) atoms and ligated carbon atoms. Hydrogen atoms were then added to fill the valencies. The hydrogen atoms were geometrically optimized keeping all heavy atoms frozen.

To model the antiferromagnetic coupling between the iron atoms within the ISCs, we used the broken symmetry (BS)[11] method by first employing the fragment sub-directive under the guess directive within Gaussian. This directive allows one to specify the α and β spins separately for unpaired electrons in each fragment. All

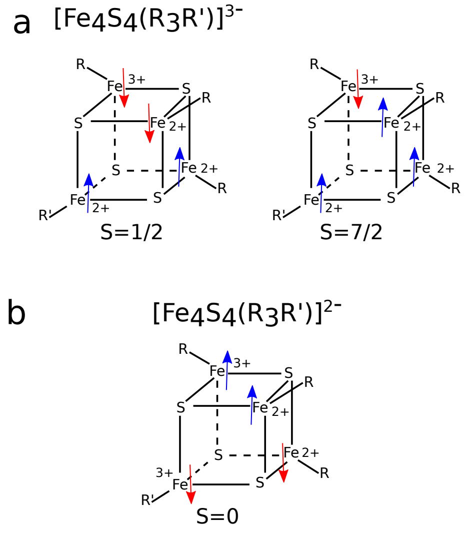

FIG. S2. Schematic diagrams of the formal iron charges and spin configurations for the iron-sulfur clusters in complex I, in both the (a) reduced and (b) oxidized states. Within the protein matrix, the ligands R/R' are cysteine residues for the iron-sulfur clusters N4 and N6a, while for N5, the ligand R' is a histidine. For the quantum calculations performed here, R/R' were SCH_3 in place of the cysteine residues for N4 and N6a and R' was NH_3 in place of the histidine residue in N5. Due to the slight asymmetry adopted in the geometry from the original PDB file, quantum mechanical calculations were performed using initial guess Hartree-Fock wavefunctions from all allowed permutations of the charge/spin configurations.

ground and excited state calculations were performed using the BS ZINDO technique.[12,13] In this methodology, the initial guess wave function is found by matching the basis set used in ZINDO with the guess and fragment keywords in Gaussian and then proceeding to the SCF and the excited state calculations.

Since the redox properties of the iron-sulfur clusters are dependent on both the Fe and S atoms, we kept the Fe and inorganic S atoms as separate fragments in the initially created guess Hartree-Fock wavefunction. Therefore, a total of twelve separate fragments were used in the QM calculations; the four iron atoms, the four inorganic sulfur atoms, and the four remaining ligands (SCH_3/NH_3). For all QM calculations, the inorganic sulfur atoms were taken in the state with a formal charge of -2 and with a spin multiplicity of 1, while the charge and spin multiplicity of SCH_3 (NH_3) ligands were taken to be -1 and 1 (0 and 1), respectively.

The transition metal iron atom is typically found to occur in one of three possible oxidations states (+2, +3, or +4) with various possible spin states (Fig. S2). The iron atoms within the tetranuclear iron sulfur clusters in complex I are found to exist under physiological conditions in two redox states with formal iron charges (+3,+3,+2,+2) and (+3,+2,+2,+2).[14] The oxidized (Ox) state consists of two ferric (+3) and two ferrous (+2) atoms anti-

ferromagnetically coupled and forming a complex with total spin $S = 0$. The reduced state has a single ferric iron atom and three ferrous atoms with an effective spin $S = 1/2$. The $S = 7/2$ reduced state has a comparable low lying ground state energy.[15] This $S = 7/2$ reduced state configuration was also included in the calculation even though it has been shown that the $S = 1/2$ state is slightly lower in energy.[15] It was also shown that the four iron atoms within tetranuclear clusters were indistinguishable via the Mössbauer spectroscopy.[16]

The polarizabilities for the reduced and oxidized states of the iron-clusters can be found by using the excited state transition energies and the transition dipole moments all of which are provided within the ZINDO framework of Gaussian'09 and the BS-ZINDO methodology used here.[12,13] Explicitly, the polarizability tensor for a given structure can be calculated using

$$\alpha_0^{\beta\gamma} = 2\sum_{j>0} \frac{\mu_{0j}^{\beta}\mu_{j0}^{\gamma}}{\Delta E_{0j}}\,, \tag{S1}$$

where $\Delta E_{0j} = E_j - E_0$ is the difference in energy between excited state j and the ground state and β, γ represent Cartesian coordinates. The scalar polarizibilities reported in Fig. S3 are traces of the corresponding tensors $\alpha_0 = \frac{1}{3}\mathrm{Tr}\,[\boldsymbol{\alpha}_0]$. The polarizabilities shown in Fig. S3 correspond to SCFs produced for the N5 cofactor for different initial configurations. The red points within the figure are polarizabilities calculated for the dominant spin state along the classical trajectory, which is determined from to the lowest energy eigenvalue of the complete Hamiltonian.

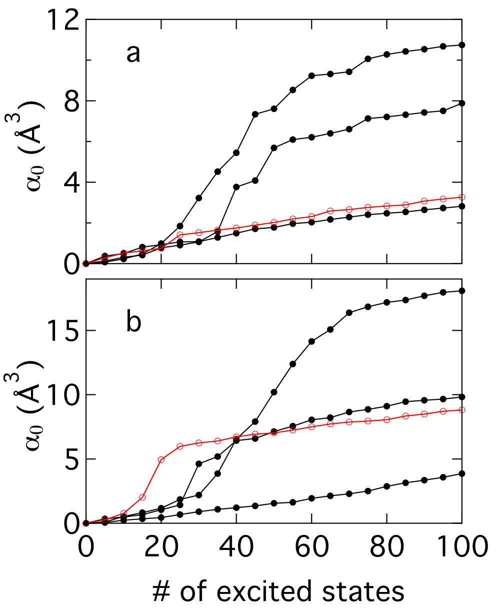

FIG. S3. The polarizability of the iron-sulfur cluster vs the number of excited states included in the sum in Eq. (S1) for the reduced (a) and oxidized (b) states of the N5 cofactor using the BS-ZINDO methodology.[12,13] Different spin states are indicated by black points (the solid lines connecting the points are drawn to guide the eye). The red points correspond to the polarizability for the dominant spin state found from the minimum energy eigenvalue of the complete Hamiltonian.

For comparison, we calculated the polarizabilities for the cofactors using the double and triple zeta basis sets (cc-pDVZ and cc-pTVZ) which include polarization functions and the full configuration interaction singles (CIS) method included in Gaussian along with the broken symmetry framework described in the main text under the Methods section (designated here as BS-CIS). One can see in Fig. S4 that the dominant spin state contribution (shown by the red open points) is very slowly converging. Because of the computational overhead (large amounts of memory and very long computational times) needed for the full CIS calculations, slowly converging polarizabilities, and the quality of the results provided by BS-ZINDO for a fraction of the computational cost, the BS-ZINDO calculation was used for all main results in this study. The full energy gap calculation was also carried out using this methodology including 50 excited states in contrast to the included 100 excited states for the BS-ZINDO method. The energy gap statistics (Stokes shift and variational reorganization energies) for the BS-CIS including 50 excited states is given in Table S3 and are consistent with the BS-ZINDO calculations.

D. Water Penetration

To understand water's role in solvating the cofactors, we calculated the average number of water molecules surrounding the N6a iron-sulfur cluster along the trajectory (Fig. S5). One can see that no waters are present within a 3 Å shell when the electron is on N4 and begin to appear after the electron arrives to N5. In the final state, with the electron on N6a, the average number of waters increases on the time-scale of ~ 150 ns from nearly 0 to ~ 6 waters. This increase in hydration corresponds to an increase in fluctuations of the electrostatic potential, as well as an increase in the fluctuations of the electric field.

E. Correlations between the cofactors

Diagonalizing the Hamiltonian matrix along the trajectory produces the lowest-energy eigensatate, with the energy $E_g^a(D/A)$, among all spin configurations in either Red or Ox state of each cofactor ($a = \mathrm{Red}, \mathrm{Ox}$). These four energies allow us to define the instantaneous energy gap[17]

$$X = \left[E_g^{\mathrm{Ox}}(D) - E_g^{\mathrm{Red}}(D)\right] - \left[E_g^{\mathrm{Ox}}(A) - E_g^{\mathrm{Red}}(A)\right] \tag{S2}$$

sampled along the trajectory. In Table S2, we have separated X into the donor and acceptor contributions in the variance $\langle(\delta X)^2\rangle$. This separation allows us to look at individual contributions to the total variance and at the cross term. Except for the N4$^-$ $\rightarrow$ N5 transition, the total variance of X is largely dominated by the sum of the variances of individual cofactors.

TABLE S2. Variances of the energy gap X (Eq. (S2)) produced from the QM/MM formalism. The total is the sum of the donor and acceptor components, $X = X_1 + X_2$, $X_1 = E_g^{\mathrm{Ox}}(D) - E_g^{\mathrm{Red}}(D)$, $X_2 = E_g^{\mathrm{Ox}}(A) - E_g^{\mathrm{Red}}(A)$. All energies are in eV. The mean of the values in the last column for a given reaction yields the reorganization energy λ in Table I in the main text. The QC subscript in classical calculation using the electrostatic potential ($X = \langle \Delta\phi \rangle_{QC}$) indicates that the potential was averaged over the quantum center, the cofactor in this case, as well as along the simulation trajectory.

N4⟷N5	$\beta \langle \delta X_1^2 \rangle /2$	$\beta \langle \delta X_2^2 \rangle /2$	$\beta \langle \delta X_1 \delta X_2 \rangle$	$\beta \langle \delta X^2 \rangle /2$
$N4^-$/N5 (1^-/2) (QM/MM)	1.75	1.63	1.29	2.09
Classical ($X = \langle \Delta\phi \rangle_{QC}$)				1.96
Classical ($X = \sum_i \Delta q_i \phi_i$)				2.8
N4/$N5^-$ (1/2^-) (QM/MM)	2.30	2.20	-0.08	4.58
Classical ($X = \langle \Delta\phi \rangle_{QC}$)				3.40
Classical ($X = \sum_i \Delta q_i \phi_i$)				6.2
N5⟷N6a	$\beta \langle \delta X_1^2 \rangle /2$	$\beta \langle \delta X_2^2 \rangle /2$	$\beta \langle \delta X_1 \delta X_2 \rangle$	$\beta \langle \delta X^2 \rangle /2$
$N5^-$/N6a (1^-/2) (QM/MM)	2.20	1.21	0.68	2.72
Classical ($X = \langle \Delta\phi \rangle_{QC}$)				2.18
Classical ($X = \sum_i \Delta q_i \phi_i$)				4.9
N5/$N6a^-$ (1/2^-) (QM/MM)	1.79	2.81	0.65	3.95
Classical ($X = \langle \Delta\phi \rangle_{QC}$)				2.97
Classical ($X = \sum_i \Delta q_i \phi_i$)				6.6

TABLE S3. The Stokes reorganization energy, $\lambda^{\mathrm{St}} = |X_{01} - X_{02}|/2$, and the variance reorganization energy, $\lambda = \sigma_X^2/(2k_BT)$, produced from the QM/MM formalism using the BS-CIS method. All energies are in eV.

Reaction	λ^{St}	λ
$N4^-$→N5	0.57	2.54
$N5^-$→N6a	1.24	2.63

II. Free energy of electron transfer

Here we provide the derivation of Eqs. (8) and (9) in the main text, connecting the free energy of electron transfer to the free energy of Coulomb interaction between the cofactors in the chain. We start with a general problem of electron transfer between two cofactors carrying the charges Z_D^{Ox} (at the donor) and Z_A^{Ox} (at the acceptor) in their oxidized (Ox) states. The total charge of the donor is therefore $Z_D^{\mathrm{Ox}} - 1$ in the initial state and the total charge of the acceptor is $Z_A^{\mathrm{Ox}} - 1$ in the final state (Fig. S6). The free energy of electron transfer can be written in the linear response approximation as[18]

$$\Delta G_0 = \Delta E_{\mathrm{gas}} - \tfrac{1}{2}\chi^L \mathbf{E}_2 * \mathbf{E}_2 + \tfrac{1}{2}\chi^L \mathbf{E}_1 * \mathbf{E}_1. \tag{S3}$$

Here, ΔE_{gas} is the gas-phase energy gap and χ^L is the longitudinal response function of the medium, which generally can be given as a non-local kernel.[19] The electric field of the donor-acceptor complex $\mathbf{E}_i$ in the initial ($i = 1$) and final ($i = 2$) states is integrated over the volume occupied by the thermal bath (protein and water). The asterisks notation involves both the volume integral and the scalar product

$$\mathbf{E}_i * \mathbf{E}_i = \int_V \mathbf{E}_i \cdot \mathbf{E}_i d\mathbf{r}. \tag{S4}$$

The fields in the initial and final states can be represented as sums of the corresponding donor and acceptor fields

$$\begin{aligned} \mathbf{E}_1 &= \mathbf{E}_D^{\mathrm{Red}} + \mathbf{E}_A^{\mathrm{Ox}}, \\ \mathbf{E}_2 &= \mathbf{E}_D^{\mathrm{Ox}} + \mathbf{E}_A^{\mathrm{Red}}, \end{aligned} \tag{S5}$$

where $\mathbf{E}_{A,D}^{\mathrm{Ox,Red}}$ are the fields of the donor (D) and acceptor (A) in the corresponding redox states. After some manipulations, we obtain

$$\Delta G_0 = \Delta E_{\mathrm{gas}} + \Delta G_s^A - \Delta G_s^D + \chi^L \left[\mathbf{E}_A^{\mathrm{Ox}} * \mathbf{E}_D^e - \mathbf{E}_D^{\mathrm{Ox}} * \mathbf{E}_A^e \right], \tag{S6}$$

where $\mathbf{E}_{D,A}^e$ is the electric field of the electron localized either on the donor or the acceptor. Further, $\Delta G_s^{D,A}$ are the solvation free energy differences between the Red and Ox states. By using the Coulomb law, this equation can be transformed to

$$\Delta G_0 = \Delta E_{\mathrm{gas}} + \Delta G_s^A - \Delta G_s^D + \frac{4\pi e^2 \chi^L}{R_{DA}} (Z_D^{\mathrm{Ox}} - Z_A^{\mathrm{Ox}}), \tag{S7}$$

where R_{DA} is the donor-acceptor distance. One has additionally to keep in mind that the free energy of the electrostatic field of the donor acceptor complex is contained in ΔE_{gas}

$$\Delta E_{\mathrm{gas}} = \Delta E'_{\mathrm{gas}} + \tfrac{1}{8\pi}\mathbf{E}_2 * \mathbf{E}_2 - \tfrac{1}{8\pi}\mathbf{E}_1 * \mathbf{E}_1. \tag{S8}$$

Combining these terms together one gets

$$\begin{aligned} \Delta G_0 = \Delta E_{\mathrm{gas}} &+ \Delta G_s^A - \Delta G_s^D \\ &+ \frac{e^2}{R_{DA}} (4\pi\chi^L - 1)(Z_D^{\mathrm{Ox}} - Z_A^{\mathrm{Ox}}), \end{aligned} \tag{S9}$$

where ΔE_{gas} is now the difference of the gas-phase energies of the isolated donor and acceptor fragments, excluding their Coulomb interaction. The standard assignment of the longitudinal susceptibility is[19] $4\pi\chi^L =$

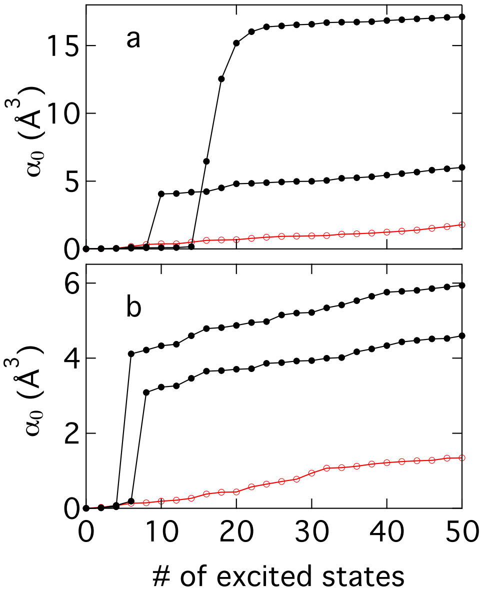

FIG. S4. The polarizability of the iron-sulfur cluster vs the number of excited states included in the sum in Eq. (S1) for the reduced (a) and oxidized (b) states of the N5 cofactor using the BS-CIS methodology. Different spin states are indicated by black points (the solid lines connecting the points are drawn to guide the eye). The red points correspond to the polarizability for the dominant spin state found from the minimum energy eigenvalue of the complete Hamiltonian.

$1 - \epsilon_{\text{eff}}^{-1}$, where ϵ_{eff} is the effective dielectric constant of the medium. One obtains the final expression

$$\Delta G_0 = \Delta E_{\text{gas}} + \Delta G_s^A - \Delta G_s^D - \frac{e^2}{\epsilon_{\text{eff}} R_{DA}} (Z_D^{\text{Ox}} - Z_A^{\text{Ox}}). \quad \text{(S10)}$$

Expressing the first three terms in this equation in terms of the standard (midpoint) reduction potentials of the donor and acceptor, one arrives at Eq. (7) in the main text. For charge separation reactions, $Z_D^{\text{Ox}} = 1$ and $Z_A^{\text{Ox}} = 0$ and one arrives from Eq. (S10) at the standard Rehm-Weller result used in the literature.[20]

The effective dielectric constant ϵ_{eff} in Eq. (S10) is not known and this relation is not practical for most heterogenous systems. An alternative can be thought in finding the interaction energy between the charges directly from simulations.

The free energy of two charges placed in a polarizable medium can be written as the sum of the direct Coulomb interaction between them and the variance of the medium potentials ϕ_1 and ϕ_2 at the location of the charges (linear response approximation[21])

$$G_s = \frac{q_1 q_2}{R} - \frac{\beta}{2} \langle (q_1 \delta\phi_1 + q_2 \delta\phi_2)^2 \rangle. \quad \text{(S11)}$$

The self variances in this equation $\langle (\delta\phi_i)^2 \rangle$ produce solvation free energies and the cross variance is responsible

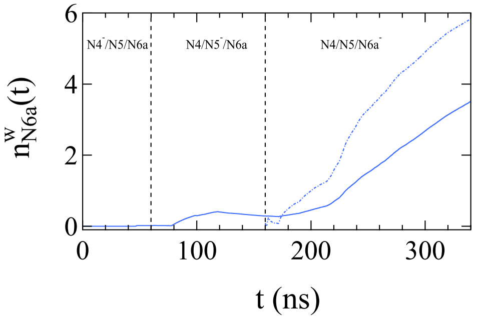

FIG. S5. The number of waters within a 3 Å shell surrounding N6a along the simulation trajectory. The solid blue curve represents the rolling average number of waters surrounding N6a from the configuration with the electron on N4 (first vertical dashed line), to the electron on N5 (second vertical dashed line), to the final state studied here with the electron on N6a. The dashed blue line represents the rolling average taken only along the trajectory with the electron on N6a.

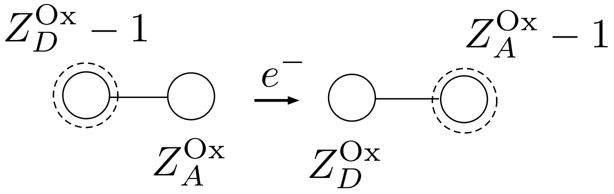

FIG. S6. Schematics of electron transfer in a donor-acceptor complex. The charged of the oxidized state are Z_D^{Ox} for the donor and Z_A^{Ox} for the acceptor.

for the potential of mean force, i.e., the alteration of the free energy of bringing the charges from infinity to the distance R

$$\Delta G_s(R) = q_1 q_2 \left[R^{-1} - \beta \langle \delta\phi_1 \delta\phi_2 \rangle \right]. \quad \text{(S12)}$$

The results of calculations of cross-variances of the medium electrostatic potentials at the cofactors of the charge-transfer chain are listed in Table S4. These results are combined with distances listed in Table S1 to calculate the profile of reaction free energies along the chain shown in Fig. 5 in the main text.

[1] Berrisford, J. M. & Sazanov, L. A. Structural Basis for the Mechanism of Respiratory Complex I. *J. Biol. Chem.* **284**, 29773–29783 (2009).

[2] Humphrey, W., Dalke, A. & Schulten, K. Vmd - visual molecular dynamics. *Journal of Molecular Graphics* **14**, 33–38 (1996).

[3] Klauda, J. B. *et al.* Update of the charmm all-atom additive force field for lipids: Validation on six lipid types. *J. Phys. Chem. B* **114**, 7830–7843 (2010).

[4] Freddolino, P. L., Dittrich, M. & Schulten, K. Dynamic switching mechanisms in lov1 and lov2 domains of plant phototropins. *Biophysical journal* **91**, 3630–3639 (2006).

[5] Freddolino, P. L., Gardner, K. H. & Schulten, K. Signaling mechanisms of lov domains: new insights from molecular dynamics studies. *Photochem. Photobiol. Sci.* (2013).

TABLE S4. Cross correlations of the fluctuations of the electrostatic potentials at the iron-sulfur clusters (V/e).

Configuration	$\beta\langle\delta\phi_I\delta\phi_{II}\rangle$	$\beta\langle\delta\phi_{II}\delta\phi_{III}\rangle$	$\beta\langle\delta\phi_I\delta\phi_{III}\rangle$)
$N4^-/N5/N6a$ ($I^-/II/III$)	1.11	1.25	0.91
$N4/N5^-/N6a$ ($I/II^-/III$)	0.29	0.60	0.02
$N4/N5/N6a^-$ ($I/II/III^-$)	1.03	0.65	0.44

[6]Pavelites, J. J., Gao, J., Bash, P. A. & Mackerell, A. D. A molecular mechanics force field for NAD+ NADH, and the pyrophosphate groups of nucleotides. *J. Comput. Chem.* **18**, 221–239 (1997).

[7]MacKerell, A. D. *et al.* All-atom empirical potential for molecular modeling and dynamics studies of proteins. *The Journal of Physical Chemistry B* **102**, 3586–3616 (1998).

[8]Chang, C. H. & Kim, K. Density Functional Theory Calculation of Bonding and Charge Parameters for Molecular Dynamics Studies on [FeFe] Hydrogenases. *J. Chem. Theory Comput.* **5**, 1137–1145 (2009).

[9]Frisch, M. J. *et al.* Gaussian09 Revision E.01. Gaussian Inc. Wallingford CT 2009.

[10]Phillips, J. C. *et al.* Scalable molecular dynamics with namd. *Journal of Computational Chemistry* **26**, 1781–1802 (2005).

[11]Noodleman, L. & Case, D. A. Density-functional theory of spin polarization and spin coupling in iron-sulfur clusters. *Advances in Inorganic Chemistry* **38**, 423–470 (1992).

[12]Hayashi, T. & Stuchebrukhov, A. A. Electron tunneling in respiratory complex I. *Proc. Natl. Acad. Sci. USA* **107**, 19157–19162 (2010).

[13]Hayashi, T. & Stuchebrukhov, A. A. Quantum Electron Tunneling in Respiratory Complex I. *J. Phy. Chem. B* **115**, 5354–5364 (2011).

[14]Oshnishi, T. Iron-sulfur clusters/semiquinones in complex i. *Biochim Biophys Acta.* **1364**, 186–206 (1998).

[15]Torres, R. A., Lovell, T., L., N. & Case, D. A. Density functional and reduction potential calculations of fe_4s_4 clusters. *Journal of the American Chemical Society* **127**, 1923–1936 (2003).

[16]Trautwein, A. Iron-containing proteins and related analogs - complementary mossbauer, epr, and magnetic-susceptibility studies. *Struct. Bonding* **78**, 1–95 (1991).

[17]Blumberger, J. & Sprik, M. Quantum vs classical electron transfer energy as reaction coordinate for the aqueous Ru^{2+}/Ru^{3+} redox reaction. *Theor. Chem. Acc.* **115**, 113–126 (2006).

[18]Milischuk, A. A., Matyushov, D. V. & Newton, M. D. Activation entropy of electron transfer reactions **324**, 172–194 (2006).

[19]Matyushov, D. V. Solvent reorganization energy of electron transfer in polar solvents. *J. Comp. Phys.* **120**, 7532–7556 (2004).

[20]Rehm, D. & Weller, A. Kinetics of fluorescence quenching by electron and H-atom transfer. *Israel J. Chem.* **8**, 259–271 (1970).

[21]Hummer, G., Pratt, L. R. & Garcia, A. E. Free energy of ionic hydration. *J. Phys. Chem.* **100**, 1206–1215 (1996).
